# Initial Evaluation of Feasibility and Cutaneous Toxicity of Electron FLASH Radiotherapy Using a Standard-of-Care Fractionation Scheme in a Porcine Skin Model

**DOI:** 10.3390/cancers18061009

**Published:** 2026-03-20

**Authors:** Elise Konradsson, Kevin Liu, Safee Baig, Susanne Je-Han Lin, Alan Hernandez Lopez, Brett Velasquez, Stephanie Mayor, Kayla Samuel, Traci Viscarra, Krystal Garrow, Erica J. Moore, William Norton, Jody Swain, Ziyi Li, Albert C. Koong, Steven H. Lin, Emil Schüler, Devarati Mitra

**Affiliations:** 1 Department of Radiation Physics, The University of Texas MD Anderson Cancer Center, Houston, TX 77030, USA; 2 Department of Veterinary Medicine and Surgery, The University of Texas MD Anderson Cancer Center, Houston, TX 77030, USA; 3 Department of Biostatistics, Division of Basic Science Research, The University of Texas MD Anderson Cancer Center, Houston, TX 77030, USA; 4 Department of Radiation Oncology, The University of Texas MD Anderson Cancer Center, Houston, TX 77030, USA

**Keywords:** ultra-high dose rate, FLASH, electron, large animal, normal tissue

## Abstract

**Background/Objectives:** FLASH radiotherapy (RT) has shown potential to reduce normal tissue toxicity compared with conventional (CONV) RT while maintaining tumor control. FLASH RT is characterized by ultra-high dose rate delivery, commonly using mean dose rates ≥ 40 Gy/s and sub-second delivery times. Most preclinical studies have used single-fraction regimens, leaving the feasibility and normal tissue impact of clinically relevant fractionation largely unexplored. We evaluated electron FLASH RT given in a standard five-fraction regimen to a porcine skin model, simulating adjuvant treatment workflow for high-risk cutaneous melanoma. **Method:** Three Yorkshire–Landrace swine received paired five-fraction electron irradiations to dorsolateral skin using either FLASH RT (mean dose rates 175–246 Gy/s) or CONV RT (8 Gy/min). Radiation was delivered with a 9-MeV electron beam; field diameters of 4, 7, or 10 cm; and doses of 5 × 6, 5 × 7, or 5 × 8 Gy. Dosimetry was validated with several dosimeters and real-time beam monitoring, confirming dose accuracy within 3%. Skin toxicity was assessed over 22–24 weeks using clinical grading, erythema spectrophotometry, and histopathologic evaluation. **Results:** FLASH RT was well tolerated at 5 × 6 Gy and 5 × 7 Gy, with no significant differences in peak radiation dermatitis, erythema index, or histologic damage compared with CONV RT. At 5 × 8 Gy, both modalities caused unacceptable toxicity, including moist desquamation and necrosis. No volume-dependent effects were observed. **Conclusions:** Although a FLASH-specific normal tissue sparing effect was not observed, this study demonstrates the technical feasibility and safety of delivering fractionated electron FLASH RT in a large animal model using a clinically relevant workflow. These findings support further investigation of physical beam parameters and biological modifiers, such as tissue oxygenation, and inform the clinical translation of fractionated FLASH RT for cutaneous malignancies.

## Simple Summary

FLASH radiotherapy delivers radiation at ultra-high dose rates and has been proposed to reduce normal tissue toxicity compared with conventional dose rate treatment. Because most evidence comes from single-fraction studies, we evaluated a clinically relevant five-fraction regimen in a porcine skin model using a workflow designed to simulate adjuvant treatment for high-risk cutaneous melanoma. Skin injury was assessed by longitudinal clinical grading, objective erythema measurements, and histopathology. Fractionated FLASH radiotherapy was technically feasible and well tolerated at clinical doses, with skin reactions comparable to conventional irradiation. No FLASH normal tissue sparing was observed in this five-fraction setting. These results support the feasibility of fractionated FLASH delivery while motivating further investigation of the biological and physical conditions required to achieve normal tissue sparing.

## 1. Introduction

FLASH radiotherapy (RT) is an emerging modality characterized by ultra-high dose rate delivery, typically with a mean dose rate ≥ 40 Gy/s [1,2]. By delivering radiation several orders of magnitude faster than standard clinical dose rates, this near-instantaneous delivery has the potential to improve the safety and tolerability of RT by mitigating intrafractional motion (potentially allowing reduced margins and thereby sparing adjacent normal tissues) and significantly shortening treatment time, improving patient comfort. In addition, preclinical studies have demonstrated that FLASH RT has the potential to selectively spare normal tissue, relative to conventional (CONV) RT, while maintaining equivalent tumor control [1,2,3,4,5,6,7,8], a phenomenon known as the FLASH effect. Beyond mean dose rate alone, several studies have suggested that other beam parameters, such as instantaneous dose rate, dose per pulse (DPP), and pulse structure, may critically influence the magnitude and consistency of the FLASH effect [9,10,11]. However, the physical and biological parameters required to reliably elicit and optimize this FLASH effect are poorly understood.

Most preclinical evidence for FLASH RT’s normal tissue sparing in skin has been generated using high single-fraction doses, typically in the range of 20–34 Gy, where robust differences between FLASH and CONV irradiation have been observed. For example, Soto et al. demonstrated that electron FLASH RT delivered at 180 Gy/s significantly reduced both the frequency and severity of skin ulceration in mice compared with CONV RT following single-fraction hemi-thoracic irradiation ≥ 30 Gy, when assessed 8 weeks post-treatment [7]. Kristensen et al. reported a dose-modifying-factor of 1.45–1.54 for single-fraction electron FLASH vs. CONV RT in a mouse hindleg model [12]. However, data on FLASH RT given in fractionated regimens remain limited [13,14], and a key question for clinical translation is whether this biological benefit of FLASH requires large single-fraction doses or can be achieved using more clinically standard fractionated regimens with lower doses per fraction.

Despite promising results in small animal models [1,2,3,4,5,6,7,8,10,11,12,13,14], translation of novel FLASH RT dose-fractionation regimens to patients requires validation in large animal systems that more closely replicate human anatomy and physiology. The porcine model offers a highly relevant platform for studying radiation-induced skin toxicity. Porcine skin closely resembles human skin in terms of epidermal and dermal thickness, vascularization, and wound healing [15]. Pigs also allow use of clinically relevant field sizes and fractionation schemes, and their genetic and physiological heterogeneity provides a realistic approximation of the variability expected in patients. Previous studies have demonstrated the feasibility of using porcine skin to evaluate FLASH RT; however, these large animal investigations have interrogated single-fraction FLASH delivery. Vozenin et al. reported reduced late toxicity of single-fraction FLASH RT, compared with CONV RT, in a mini-pig treated with doses of 28–34 Gy to a circular field 2.6 cm in diameter [16]. More recent single-fraction studies in large animals using similar doses have raised concerns about severe toxicity such as fibrosis and necrosis, underscoring the need for further investigation into the clinically relevant bounds of FLASH sparing [17,18]. Notably, Rohrer Bley et al. observed volume-dependent long-term cutaneous toxicities, with larger fields (3.5 × 4.5 cm and 8 × 8 cm) associated with unacceptable late toxicity [17].

Before clinical implementation, rigorous preclinical validation is therefore needed to define the safety profile, translational relevance, and optimal delivery parameters of FLASH RT in clinically realistic scenarios. Several early-phase clinical trials, primarily focusing on safety, have already been completed or are ongoing [19,20,21]. Electron irradiation is particularly well suited for rapid clinical translation, and its limited penetration depth makes superficial tumors, such as aggressive cutaneous malignancies, an ideal clinical target. At our institution, hypofractionated electron RT is commonly used in the postoperative management of high-risk cutaneous melanoma, given in five fractions of 6 Gy each (prescribed to the depth of maximum dose [Dmax]) [22,23]. Patients with melanoma who have positive margin resection, desmoplastic histology or microsatellitosis have been reported to have >20% risk of local recurrence, which can require morbid surgical re-resection and potential for aggressive systemic therapy [24,25,26,27]. Such treatment intensification is more likely to be associated with long-term toxicity. Adjuvant RT to the cutaneous tumor bed can decrease local recurrence by >50% and serves as an important tool in the multidisciplinary care of patients with melanoma [24,25]. Nearly all patients develop grade 2 radiation dermatitis, which can be followed from onset to resolution. Accordingly, in the present study, we aimed to evaluate the toxicity profile of electron FLASH RT building from a standard-of-care five-fraction irradiation regimen. We selected fraction doses of 6–7 Gy to directly replicate clinically used regimens and included a 5 × 8 Gy cohort to define an upper clinical boundary and to test whether any dose-rate-dependent sparing would emerge at a higher per-fraction dose. Importantly, these per-fraction doses are below the single-fraction dose threshold at which FLASH sparing has been most consistently observed, but they are above doses that have been reported in some studies to still elicit a normal tissue sparing effect using lower dose per-fraction deliveries [28]. Thus, the chosen fractionation scheme enables clinically relevant translation while also allowing assessment of whether FLASH sparing persists under more modest dose conditions in the context of cutaneous irradiation.

In contrast to prior single-fraction large-animal studies, the current study provides an initial evaluation of the feasibility and cutaneous toxicity of FLASH RT vs. CONV RT delivered in a standard-of-care five-fraction regimen to large skin target volumes using a clinical workflow. By comparing FLASH irradiation and CONV irradiation under conditions that closely mimic the clinical treatment protocol for postoperative cutaneous melanoma used at our institution, this study is designed to assess the feasibility and safety under clinically translatable conditions and to determine whether a FLASH normal tissue sparing effect is detectable when using clinically relevant fractionation and field sizes.

## 2. Materials and Methods

### 2.1. Experimental Design

**Figure 1:**
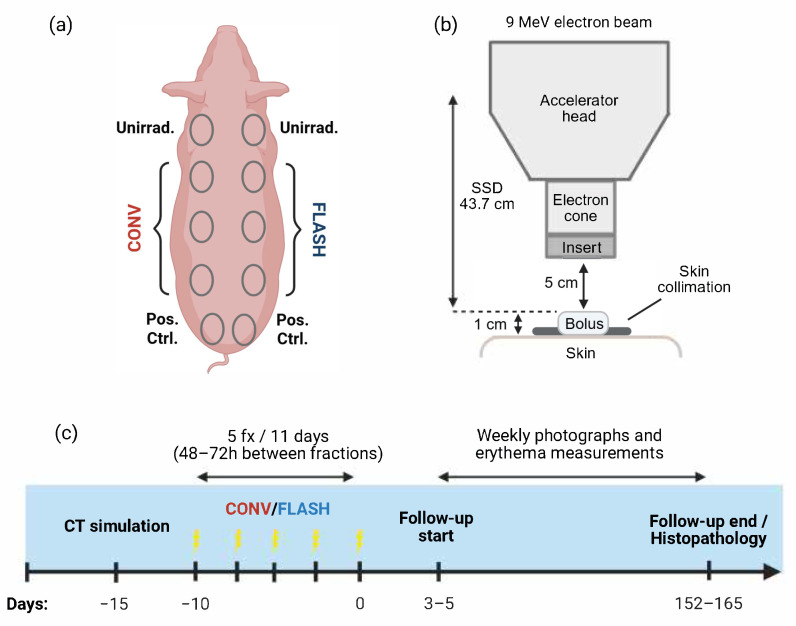
(**a**) Schematic representation of field placements, including six treatment fields, two unirradiated control fields, and two “positive control” fields per animal. (**b**) Experimental setup used for irradiations. Treatments were delivered with a 9-MeV electron beam at a source-to-surface (SSD) distance of 43.7 cm, with a 5 cm air gap, lead skin collimation, and 1 cm tissue-equivalent bolus. (**c**) Experimental timeline. Radiation was given in 5 fractions delivered over 11 days, and the animals were followed for 152–165 days after the final fraction.

To compare FLASH RT and CONV RT in a standard-of-care 5-fraction RT regimen, we irradiated three Yorkshire–Landrace swine (Oak Hill Genetics, Ewing, IL, USA) weighing 43–49 kg at the onset of radiation delivery. The swine were housed in an indoor, climate-controlled, AAALAC-accredited animal research facility at MD Anderson Cancer Center. All procedures were approved by the Institutional Animal Care and Use Committee. Six fields were delineated on each animal (3 fields per dorsolateral flank, Figure 1a), enabling 18 unique combinations of modality (FLASH or CONV), field size (4, 7, or 10 cm), and dose (5 × 6 Gy, 5 × 7 Gy, or 5 × 8 Gy). Each animal received one field of each field size and one of each dose level for both modalities. Each animal also had two paired single-fraction “positive control” (1 × 20 Gy CONV, 5 cm field) fields on the superior aspect of the left and right hindlimb, and two paired unirradiated “negative control” fields at the shoulder, to assess within- and between-animal variation. Details of the field placement are shown in Supplementary Figure S1. The experimental workflow mirrored the protocol for postoperative primary cutaneous melanoma RT at our institution, including CT simulation scans, treatment planning, machine quality assurance (QA), patient-specific QA, and longitudinal follow-up.

### 2.2. FLASH and Conventional Radiation

Radiation was delivered with a 9-MeV electron beam from a FLASH-capable linear accelerator (Mobetron, IntraOp Medical, Sunnyvale, CA, USA) [29]. All irradiations were administered at 43.7 cm source-to-surface distance, with lead skin collimation, 1 cm tissue-equivalent bolus, and a 5 cm air gap (Figure 1b). Each field received five fractions over 11 days (48–72 h intervals, Figure 1c). Radiation was delivered either at CONV dose rates (8 Gy/min) or at FLASH dose rates (175–246 Gy/s). As per the clinical standard for adjuvant melanoma RT, the dose was prescribed to Dmax such that the target volume was expected to receive 90% of the prescription dose. CONV radiation was delivered with a pulse repetition frequency (PRF) of 30 Hz and a nominal pulse width of 1.2 µs. FLASH irradiations were delivered in four pulses at a PRF of 90 Hz, with a total irradiation time of 33 ms. To ensure that the prescribed dose was achieved at the Dmax, the pulse width was adjusted accordingly. Anesthesia was induced by using tiletamine–zolazepam (4.4 mg/kg) and xylazine (2–2.2 mg/kg) and maintained with 2–4% isoflurane and 100% oxygen after intubation. This oxygenation strategy was mandated by veterinary anesthesia safety protocols for large-animal procedures at our institution to minimize hypoxemia and anesthetic risk. CONV and FLASH irradiations were performed under identical anesthetic and oxygenation conditions.

### 2.3. Beam Characterization and Dose Verification

Dose profiles and percentage depth dose (PDD) were acquired for both FLASH and CONV beams. CT simulation scans were obtained under anesthesia 5 days before delivery of the first fraction. Radiopaque skin markers and tattoos were used to outline field locations. The treatment planning system electronRT (.decimal^®^, Sanford, FL, USA) was used to simulate and visualize dose distributions. The cutaneous tissue within the field was defined as the target volume, and dose was prescribed to Dmax.

One week before the first fraction, doses were verified by using a multi-dosimeter approach. For the largest fields and at each dose level, three types of dosimeters (radiochromic film [GafChromic EBT3 film, Ashland Inc., Wayne, NJ, USA], thermoluminescent dosimeters [TLDs], and 4.8 mm diameter alanine pellets [Harwell Dosimeters Ltd., Didcot, UK]) were irradiated in the prescribed geometry (at the depth of Dmax, including skin collimation and 1 cm bolus) in triplicate, with 5 fractions used to reach the total dose, and analyzed as previously described [29]. For the smaller fields, output factors were verified with EBT3 films in triplicate with one fraction at each dose level. Daily machine and patient QA were performed using a cross-calibrated ionization chamber (Exradin A30, Standard Imaging, Middleton, WI, USA) with a 0.3 mm electrode gap, previously shown to be linear up to DPP levels of 5 Gy [30]. In vivo dose validation was performed using EBT3 film at the skin surface, extrapolated to the prescription depth (Dmax) using PDD ratios. FLASH output was also monitored in real time using a beam current transformer (BCT, Bergoz Instrumentation, Saint-Genis-Pouilly, France) [31]. The delivered dose measured by the EBT3 film and the dose estimated by the BCT were compared with the prescribed dose.

### 2.4. Acute and Late Skin Toxicity

Animals were monitored weekly from treatment start until they reached a weight of approximately 125 kg (22–24 weeks after irradiation), at which point the observation period was concluded owing to institutional housing constraints. The primary endpoint was peak radiation dermatitis, graded prospectively and weekly by a board-certified radiation oncologist using the modified RTOG radiation dermatitis grading scale (Table 1) [32], aligned with standard clinical descriptors (erythema, dry/moist desquamation, ulceration/necrosis). Erythema index was measured weekly (3 measurements per field) using a Mexameter^®^ MX18 spectrophotometer (Courage+Khazaka electronic GmbH, Köln, Germany). Erythema severity was categorized according to an erythema index scale: 0–170 (no erythema), 170–330 (minimal erythema), 330–450 (diffuse redness), 450–570 (high erythema), and >570 (extreme erythema) [33]. At the end of follow-up, skin and subcutaneous tissue specimens were subjected to histopathologic analysis. Lesions were graded by a pathologist using a semi-quantitative histopathologic grading scale (grade 0–4) that had been adapted based on general histopathologic principles (Table 1). Epidermal and dermal thicknesses were measured in hematoxylin-and-eosin-stained samples using HALO Image Analysis Platform v3.6.4134.464 (Indica Labs, Inc., Albuquerque, NM, USA), with 10 epidermal and 5 dermal sites per field.

### 2.5. Statistical Analysis

Statistical analysis was performed with GraphPad Prism version 10.3 (GraphPad Software, Boston, MA, USA) and R version 4.4.2 (R Core Team, [2024] R: R Foundation for Statistical Computing, Vienna, Austria). Normality of variables was assessed with the Shapiro–Wilk test. Because most observations deviated from normal distribution, non-parametric methods were used for groupwise comparisons. All tests were two-sided with α = 0.05.

Given the hierarchical design (multiple fields within each animal), we performed cluster-aware sensitivity analysis to address within-animal correlation by treating the animal as the unit of inference. For each endpoint, we computed animal-level summaries (median per animal × dose × treatment) and compared CONV vs. FLASH using paired Wilcoxon signed rank tests (two-sided; Pratt to include zero differences), with animal as the unit of inference. Effect sizes were the Hodges–Lehmann median within-animal difference (CONV − FLASH) with 95% confidence interval (CI). Given N = 3 animals, we emphasize effect sizes with 95% CIs over sole reliance on *p*-values.

To evaluate whether field-level values exhibited additional within-animal clustering, we used bilateral control fields (identical dose/modality/time course across animals). For all endpoints except erythema, bilateral 20 Gy CONV control fields were used. We quantified within-animal dispersion as the absolute difference between bilateral controls, |Left − Right|, for each animal, and between-animal dispersion as absolute differences on a consistent side across animals (e.g., |Animal 1 Left − Animal 2 Left|). For the erythema endpoint, baseline variability was assessed using bilateral unirradiated control fields measured weekly over the full follow-up. Within-animal comparisons were assessed with paired Wilcoxon tests, and between-animal variability was evaluated with the Kruskal–Wallis test. To assess changes in erythema over time in unirradiated fields, the Friedman test was applied to the left and right sides of each animal.

Field size effects were assessed at the dose level using Kruskal–Wallis across sizes, interpreted exploratorily given limited replication and partial confounding with animals. Pooling across field sizes for that dose/endpoint was performed only when no size effect was detected (all *p* > 0.05).

## 3. Results

### 3.1. Dosimetry

**Figure 2:**
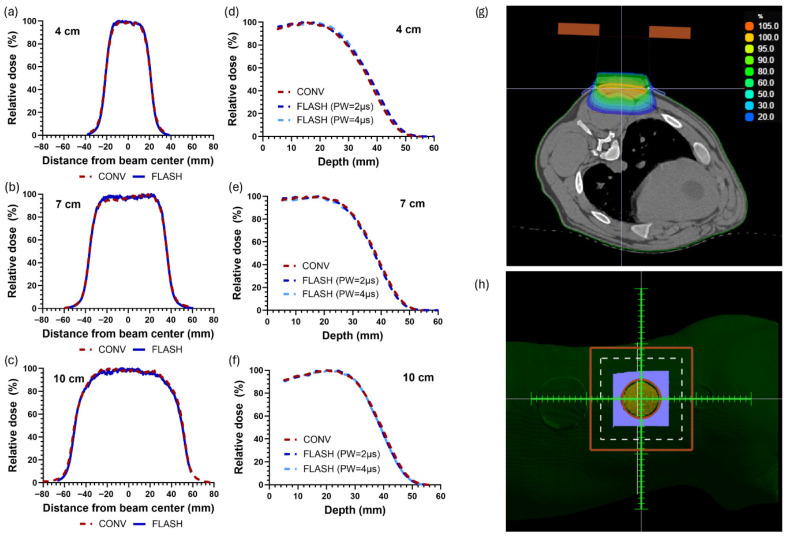
(**a**–**c**) Crossline dose profiles measured with radiochromic film placed beneath 1 cm of tissue-equivalent bolus and lead skin collimation for conventional (CONV) and FLASH irradiation. (**d**–**f**) Percentage depth dose (PDD) curves measured with radiochromic film in solid water for CONV and two FLASH beam settings with different pulse widths (PWs). The CONV and FLASH beams were matched in terms of dose distribution and demonstrated uniform dose coverage throughout the treated skin volume. (**g**) A representative transverse slice through the center of a 7 cm field (Pig 1) in the treatment planning system electronRT, with a 1 cm tissue-equivalent bolus and lead skin collimation. The skin surface within the field was defined as the target volume, and dose was prescribed to Dmax such that the target volume received ≥90% of the prescribed dose. (**h**) The corresponding beam’s-eye-view.

PDD curves and dose profiles for CONV and FLASH beams demonstrated comparable spatial distributions (Figure 2a–f). A representative treatment plan is shown in Figure 2g,h. Dmax was 1.6 cm for the 4 cm and 7 cm fields, and it was 2.2 cm for the 10 cm field. For FLASH irradiations, DPP values ranged from 1.5 to 2 Gy, with mean dose rates of 175 to 246 Gy/s and pulse dose rates ranging from 0.6 × 10^6^ to 0.7 × 10^6^ Gy/s (Table 2). For CONV irradiations, DPPs ranged from 4 to 5 mGy, mean dose rates from 0.12 to 0.14 Gy/s, and pulse dose rates from 3 × 10^3^ to 4 × 10^3^ Gy/s. Complete numerical values for each dose × field size (DPP, PRF, PW, instantaneous dose rate, total delivery time, and mean dose rate) are provided in Supplementary Table S1. The multi-dosimeter evaluation for the 10 cm field demonstrated relative standard deviations within 5% across all dose groups. Combining all dosimeter readings, the average of all measurements agreed with the prescribed dose within 3%. For the 4 cm and 7 cm fields, EBT3 film verification measurements showed an agreement with the prescribed dose within 4.3% (mean difference 1.3%) and 5% (mean difference 1.2%). Estimated delivered doses to the animals based on in vivo film dosimetry were within 3% of the prescribed total doses for all CONV and FLASH fields. For FLASH irradiations, the BCT confirmed output accuracy, with estimated delivered doses based on daily calibration factors within 2% of the prescribed values. Detailed results from the multi-dosimeter evaluation and in vivo dose measurements are provided in Supplementary Figures S2 and S3.

### 3.2. Acute Toxicity

**Figure 3:**
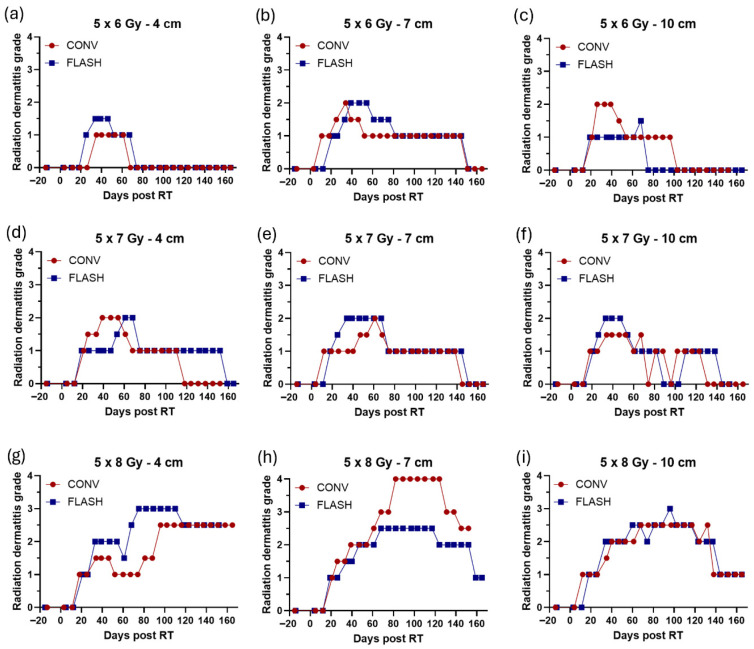
Radiation dermatitis graded longitudinally using the modified RTOG radiation dermatitis grading scale (grades 1–4) after fractionated conventional (CONV, 8 Gy/min) or FLASH (175–246 Gy/s) irradiation in porcine skin. Irradiation was delivered as 5 fractions of 6 Gy, 7 Gy, or 8 Gy using field sizes of 4 cm, 7 cm, or 10 cm. Panels (**a**–**i**) compare CONV (red circles) and FLASH (blue squares): (**a**–**c**) 5 × 6 Gy with field sizes of 4 cm, 7 cm, and 10 cm; (**d**–**f**) 5 × 7 Gy with field sizes of 4 cm, 7 cm, and 10 cm; (**g**–**i**) 5 × 8 Gy with field sizes of 4 cm, 7 cm, and 10 cm. Follow-up duration was 22–24 weeks (152–165 days) after the final fraction.

**Figure 4:**
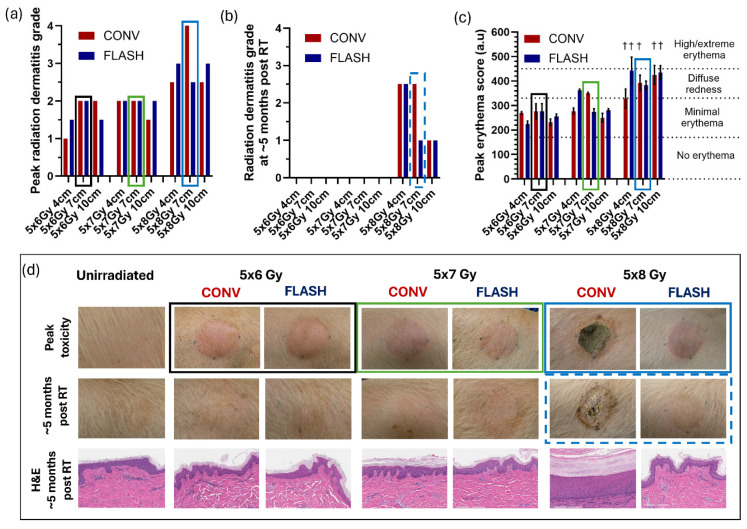
Cutaneous skin toxicity after fractionated conventional (CONV, 8 Gy/min)) or FLASH (175–246 Gy/s) irradiation in porcine skin. Irradiation was delivered as 5 fractions of 6 Gy, 7 Gy, or 8 Gy to field sizes of 4 cm, 7 cm, or 10 cm. (**a**) Peak radiation dermatitis scores and (**b**) scores at end of follow-up (152–165 days, i.e., 22–24 weeks after the final fraction) were assessed by a radiation oncologist using the modified RTOG radiation dermatitis grading scale. (**c**) Peak erythema scores (mean ± SEM) were assessed weekly throughout the follow-up period with a Mexameter MX18 spectrophotometer, and scores were calculated as the average of 3 repeated measurements per time point. † Measurements in fields with ongoing desquamation or necrosis were obtained at the field periphery, which may have led to underestimation of erythema severity. (**d**) Photographs and hematoxylin-and-eosin-stained histologic sections from 7 cm irradiated fields. Fields treated with 5 × 6 Gy (black boxes) or 5 × 7 Gy (green boxes) exhibited radiation dermatitis grade ≤ 2 and minimal erythema or, in some cases, diffuse redness. In contrast, fields receiving 5 × 8 Gy (blue boxes) showed more severe erythema responses, and resulted in patchy to confluent moist desquamation, with one field (CONV) progressing to tissue necrosis.

Initial signs of radiation dermatitis (grade 1) were observed at 2–4 weeks after the final fraction (Figure 3). Toxicity progressed over time, with peak dermatitis occurring 5 ± 1 weeks (5 × 6 Gy), 6 ± 3 weeks (5 × 7 Gy), and 12 ± 3 weeks (5 × 8 Gy) after the final fraction. Peak radiation dermatitis was significantly affected by dose level (*p* < 0.0001) but not by the volume of tissue irradiated (i.e., field size). All fields irradiated with 5 × 6 Gy or 5 × 7 Gy reached a maximum of grade 2 dermatitis, and paired animal-level analyses detected no statistical difference between CONV and FLASH (HL median within-animal difference [CONV − FLASH] at 5 × 6 Gy: 0, 95% CI −0.5 to 0.5, *p* > 0.999; 5 × 7 Gy: 0, 95% CI 0.5 to 0, *p* > 0.999; 5 × 8 Gy: 0, 95% CI −1 to 0, *p* > 0.999; Figure 4a). In contrast, fields receiving 5 × 8 Gy developed more severe injury, including patchy or confluent moist desquamation, and one field (7 cm CONV) had progressed to tissue necrosis by 12 weeks after the final fraction (Figure 4d).

The maximum erythema index was also significantly influenced by dose level (*p* < 0.0003) but not by irradiated volume. Fields irradiated with 5 × 6 Gy and 5 × 7 Gy exhibited “minimal erythema” (as characterized by the standard Mexameter MX18 scale) or, in some cases, “diffuse redness” (Figure 4c). At 5 × 8 Gy, erythema index values indicated more extensive erythema. When moist desquamation or necrosis was present, erythema was measured at the field periphery, which underestimated the true peak relative to the denuded central region. No significant difference in peak erythema index was observed between CONV and FLASH at any doses (5 × 6 Gy: HL 13, 95% CI −45 to 51), *p* = 0.75; 5 × 7 Gy: HL −12, 95% CI −24 to −4, *p* = 0.25; 5 × 8 Gy: HL −50, 95% CI −107 to 42, *p* = 0.50).

The time to onset of radiation dermatitis after treatment was not significantly affected by dose level or field size. However, dose level had a significant effect on the time to peak radiation dermatitis (*p* = 0.0005). No significant difference was observed between CONV and FLASH in time to progression or time to peak toxicity for any dose group (5 × 6 Gy—time to progression: HL 0, 95% CI −1 to 2, *p* > 0.999; time to peak: HL −2, 95% CI −5 to 0, *p* = 0.50. 5 × 7 Gy—time to progression: HL 0, 95% CI −1 to 0, *p* > 0.999; time to peak: HL 0, 95% CI 0 to 1, *p* > 0.999. 5 × 8 Gy—time to progression: HL 0, 95% CI −1 to 0, *p* > 0.999; time to peak: HL 0, 95% CI 0 to 1, *p* > 0.999). Radiation dermatitis began to resolve 8 ± 3 weeks (5 × 6 Gy), 10 ± 2 weeks (5 × 7 Gy), and 18 ± 2 weeks (5 × 8 Gy) after the last fraction, except for one 4 cm CONV field receiving 5 × 8 Gy, which showed no sign of improvement during the follow-up period (Figure 3a–i).

Full paired animal-level comparisons (Hodges–Lehmann median within-animal differences [CONV − FLASH], 95% CIs, and Wilcoxon–Pratt *p*-values) for all endpoints at each dose are provided in Supplementary Table S2.

### 3.3. Late Toxicity

At the end of follow-up (22–24 weeks after the final fraction), radiation dermatitis had fully resolved in all fields receiving 5 × 6 Gy and 5 × 7 Gy (Figure 4b). Fields receiving 5 × 8 Gy still showed grade 1–2.5 toxicity, but there were no significant differences between CONV and FLASH (*p* > 0.999).

Tissues for histologic evaluation were collected at necropsy from both the center of each field and from the periphery of the field (spanning tissue within the area of skin collimation as well as just beyond it). Morphologic changes were most prominent in the central samples, whereas peripheral samples showed milder changes that gradually normalized in the area blocked by skin collimation. Fields receiving 5 × 8 Gy were excluded from epidermal and dermal thickness analyses owing to moist desquamation and/or necrosis with lack of normal cutaneous architecture. Among fields receiving 5 × 6 Gy or 5 × 7 Gy, no significant differences in epidermal or dermal thickness were observed between CONV and FLASH at 5 × 6 Gy (epidermis: HL −1.44 µm, 95% CI −10.95 to 5.95 µm, *p* = 0.75; dermis: HL −175.1 µm, 95% CI −390.9 to 195.5 µm, *p* = 0.75) or 5 × 7 Gy (epidermis: HL −0.51 µm, 95% CI −7.66 to 0.67 µm, *p* = 0.75; dermis: HL −464.2 µm, 95% CI −738.8 to 898.6 µm, *p* > 0.999), and likewise at 5 × 8 Gy (epidermis: HL 7.14 µm, 95% CI −7.89 to 8.73 µm, *p* = 0.75; dermis: HL 4.41 µm, 95% CI −2533 to 3093 µm, *p* = 0.75)

Histopathologic analysis revealed grade 2–4 orthokeratotic hyperkeratosis, epidermal hyperplasia, dermal fibrosis, and lymphohistiocytic inflammation in some 5 × 8 Gy fields (Supplementary Figure S4). The 4 and 7 cm CONV fields receiving 5 × 8 Gy were the only fields with severe (grade 4) histologic lesions. All lower dose groups exhibited only grade 0 or 1 tissue changes. Paired animal-level estimates for epidermal and dermal thickness at each dose are detailed in Supplementary Table S2.

### 3.4. Within- and Between-Animal Comparison

Positive control fields (1 × 20 Gy CONV) developed visible radiation dermatitis within 2–3 weeks after treatment, with peak toxicity observed at 15 ± 2 weeks (Supplementary Figure S5). All positive control fields progressed to moist desquamation, and two developed necrosis.

Maximal erythema index values ranged between 349 to 471, consistent with diffuse or high-grade erythema. However, owing to the presence of moist desquamation or necrosis, some erythema measurements were acquired at the field periphery, underestimating the severity of injury. Analysis of bilateral 20 Gy CONV controls showed that within-animal absolute differences did not differ significantly from between-animal differences (all *p* > 0.05), indicating no measurable within-animal clustering beyond baseline biological variability under identical conditions.

For the erythema endpoint, baseline variability assesses using bilateral unirradiated control fields measured weekly over the full follow-up. Within-animal comparisons showed no significant difference in subjects 1 and 2 (*p* = 0.174 and *p* = 0.138) or between animals (*p* = 0.603). Subject 3 showed a significant within-animal difference (*p* = 0.019); however, all values remained below 170—the threshold for clinically meaningful erythema per the Mexameter MX18’s scale. No significant temporal changes were observed in untreated fields (*p* > 0.05).

## 4. Discussion

As a step toward informing our institution’s first clinical trial of FLASH RT, this study provides an initial evaluation of the safety and cutaneous toxicity of FLASH RT delivered in a standard-of-care hypofractionated irradiation regimen in a porcine skin model. Under the tested conditions and beam parameters, FLASH irradiation was equally well-tolerated as CONV irradiation at the standard-of-care dose levels (5 × 6 Gy and 5 × 7 Gy [34,35]), with no significant differences in acute or late skin toxicity. At the highest dose level (5 × 8 Gy), both modalities produced what would be clinically interpreted as unacceptable toxicity, including persistent moist desquamation and, in one case, tissue necrosis. Collectively, these findings indicate that five-fraction FLASH irradiation, delivered using the beam parameters of the current study to ≥4 cm cutaneous targets, produces skin toxicity similar to CONV irradiation, and that 5 × 8 Gy lies outside a clinically acceptable range in this model.

Although numerous preclinical studies have demonstrated the potential of FLASH RT to reduce normal tissue toxicity relative to CONV RT [1,2,3,4,5,7,8,10,11,12,13,14], most have used single or few-fraction regimens with limited clinical precedence for cutaneous targets. The literature on fractionated FLASH RT remains sparse but is gradually emerging. Montay-Gruel et al. demonstrated that hypofractionated FLASH RT (3 × 10 Gy) was effective in treating glioblastoma in mice while significantly reducing neurocognitive side effects compared with CONV RT [13], suggesting that the FLASH sparing effect can be preserved across multiple fractions in the central nervous system. However, Kristensen et al. reported that the skin sparing seen with single-fraction FLASH irradiations in the hindleg of CDF1 mice [12] was increasingly lost in fractionated treatments, with almost no FLASH sparing observed when eight fractions were used [14]. These findings support the notion that fractionation might negate the FLASH sparing effect, although the optimal delivery parameters remain to be defined. In this context, our work extend prior studies by evaluating a clinically relevant five-fraction FLASH regimen in a large animal skin model, providing important translational insights.

A key strength of this study is the use of the Yorkshire–Landrace swine model, which closely mimics human cutaneous biology (including epidermal and dermal thickness, vascular architecture, and wound healing dynamics), allows testing of clinically relevant target field sizes, and captures some of the genetic diversity expected in cancer patients [15].

Another major strength of this study was its clinically realistic workflow, closely mirroring the protocol for postoperative cutaneous melanoma at our institution, including machine QA, CT simulation, treatment planning, patient-specific QA, and longitudinal follow-up. Robust dosimetry verification confirmed dose accuracy and delivery reliability. Importantly, PDDs and beam profiles were matched between FLASH and CONV beams, isolating dose rate as the only variable and strengthening the validity of our comparison. Our delivered FLASH beam parameters (Table 2) fall within the operational ranges commonly associated with normal tissue sparing in prior preclinical reports—namely, DPP ≥ 1 Gy/pulse, instantaneous dose rates in the hundreds of kGy/s to MGy/s, mean dose rates ≥ 40 Gy/s, and total delivery durations < 100 ms [1,2,9,10,11]. The threshold for fraction dose remains less well defined. While the 6–8 Gy/fraction used in our study reflects clinical practice for cutaneous malignancies and lies below the ≥10 Gy single-fraction threshold often associated with robust sparing in small animal models [8,36], it has been reported that the sparing effect can be maintained under lower dose-per-fraction deliveries (3 Gy/fraction) [28].

Our study also contributes to the growing body of literature evaluating FLASH RT in large animal models, particularly in the context of skin toxicity. Previous single-fraction FLASH studies in pigs and veterinary patients have had mixed outcomes regarding safety and long-term toxicity [16,17,18,37,38]. Vozenin et al. first demonstrated the potential of FLASH RT to reduce normal tissue toxicity in mini-pigs and feline cancer patients [16], whereas subsequent work identified dose- and volume-dependent limitations, including fibrosis and necrosis [17,18]. In contrast, our study did not detect a volume-dependent effect on skin toxicity across the tested field sizes (4, 7, and 10 cm), within a five-fraction regimen at clinically relevant doses, although this study was underpowered to detect small-to-moderate volume differences, and architectural loss at 5 × 8 Gy precluded some quantitative analyses. Subtle size-related effects, therefore, cannot be excluded.

It should be noted that when moist desquamation or necrosis occurred, erythema measurements were obtained at the field periphery, likely underestimating peak severity and biasing dose–response relationships and modality comparisons toward the null. Although this approach was applied uniformly to FLASH and CONV fields—preserving internal validity—absolute erythema values, particularly at 5 × 8 Gy, should be interpreted conservatively.

Another important consideration in interpreting our results is the use of 100% oxygen ventilation under isoflurane anesthesia, mandated by veterinary anesthesia safety protocols for large-animal procedures at our institution to minimize hypoxemia and anesthesia-related complications. Hyperoxia has been reported in murine models to diminish or even abolish the FLASH sparing effect [39,40,41,42,43], suggesting that our operating conditions could have mitigated the protective effects of FLASH irradiation in normal tissue. To address this, future studies will explore the influence of oxygen modulation in large animal models to better understand its role in mediating the FLASH effect, which might be essential for optimizing clinical protocols.

Beyond the biological considerations, our study also had practical limitations. The rapid growth of the Yorkshire–Landrace swine breed limited the follow-up period to approximately 5.5 months, as the animals eventually outgrew the capacity of the housing facilities and treatment equipment. While sufficient for acute/subacute dermatitis and early remodeling, this horizon may under-detect late fibrosis/atrophy, which can continue to evolve beyond 6–12 months. Moreover, given the small cohort (N = 3) and multiple comparisons across dose, field size, and endpoints, results are best interpreted as exploratory, with emphasis on effect sizes and 95% CIs rather than sole reliance on *p*-values. However, this study was not designed to provide statistical evidence of a FLASH sparing effect but rather to perform an initial evaluation under conditions that closely mimic the clinical treatment protocol for postoperative cutaneous melanoma at our institution.

Importantly, the absence of a detectable FLASH sparing effect under these conditions does not negate the FLASH effect but rather constrains the regimen- and context-specific parameter space in which sparing may occur. Our data directly support toxicity equivalence under matched dose distributions in a clinically realistic, fractionated regimen and demonstrate that FLASH RT is safe, feasible, and well-tolerated in a large animal cutaneous model. Even without a clear biological sparing effect, FLASH RT offers practical advantages that may benefit clinical workflows, including ultra-short delivery times that may reduce intrafraction motion and improve patient comfort. Moreover, the ultra-fast FLASH RT delivery could spare circulating immune cells that transiently pass through the radiated field, which could alter the dynamics of the acute inflammatory response [44,45]. This feature could enhance the synergy between RT and immune checkpoint inhibitors, already a key interest area for cancers like melanoma for which such inhibitors have become standard treatment [46].

## 5. Conclusions

Overall, our findings indicate that FLASH irradiation is as safe and well tolerated as CONV irradiation when delivered using a standard-of-care hypofractionated regimen in a porcine skin model that closely mimics human cutaneous skin. This study provides important preclinical evidence supporting the clinical feasibility of fractionated FLASH RT and validates a robust, clinically relevant workflow and dosimetry protocol that can be directly applied to early-phase clinical trials. Further investigations are warranted to optimize FLASH delivery parameters and to better define the conditions under which normal tissue sparing can be achieved.

## Figures and Tables

**Table 1:** Grading scales for radiation dermatitis and histopathologic assessment.

	Grade 0	Grade 1	Grade 1.5	Grade 2	Grade 2.5	Grade 3	Grade 4
RTOG modified Radiation Dermatitis Scale	No change over baseline	Follicular, faint or dull erythema	Dry desquamation	Tender or bright erythema	Patchy moist desquamation	Confluent moist desquamation	Ulceration, hemorrhage, necrosis
Histopathology	No tissue change compared to unirradiated area	Minimal, minor, rare, infrequent, barely noticeable tissue change	-	Mild, slight, sporadic, noticeable but not prominent feature	-	Moderate, frequent, typical, common, prominent tissue change	Marked, extensive, numerous, severe, overwhelming tissue change

**Table 2:** Summary of delivered beam parameters.

	FLASH	CONV
Mean dose rate (Gy/s)	175–246	0.12–0.14
Dose per pulse, DPP (Gy/pulse)	1.5–2.0	0.004–0.005
Pulse repetition frequency, PRF (Hz)	90	30
Pulses per fraction	4	1240–1990
Pulse width (µs)	2.3–3.4	1.2
Pulse dose rate (MGy/s)	0.58–0.69	0.003–0.004
Total delivery time per fraction	33 ms	minutes

## Data Availability

The data presented in this article will be made available by the authors upon reasonable request.

## Supplementary Materials

The following supporting information can be downloaded at: https://www.mdpi.com/article/10.3390/cancers18061009/s1, Figure S1: Illustration of the field placements on each animal; Figure S2: Pretreatment dose verification in the prescribed geometry, i.e., at the depth of dose maximum, including skin collimation and 1 cm bolus; Figure S3: In vivo dose validation during animal irradiations; Figure S4: Histopathologic evaluation of porcine skin after fractionated conventional (CONV; 8 Gy/min) or FLASH (175–246 Gy/s) irradiation; Figure S5: Radiation dermatitis graded over time using the modified RTOG radiation dermatitis grading scale (grades 1–4) for each of the six positive control fields (left and right side of each animal) irradiated with 1 × 20 Gy CONV RT (5-cm field size); Table S1: Physical beam parameters used for FLASH and CONV irradiations; Table S2: Paired comparisons were performed at the animal level using the Wilcoxon signed‑rank test (two‑sided; Pratt). Effect sizes are reported as Hodges–Lehmann (HL) median within‑animal differences (CONV − FLASH) with 95% confidence intervals (CI).
