# Supplementary material for: Initial Evaluation of Feasibility and Cutaneous Toxicity of Electron FLASH Radiotherapy Using a Standard-of-Care Fractionation Scheme in a Porcine Skin Model
Source: Cancers (Basel). 2026 Mar 20;18(6):1009. doi: 10.3390/cancers18061009 (PMC13024774; doi:10.3390/cancers18061009)
Supplement: Supplementary file 1 [file cancers-18-01009-s001.zip › cancers-4175452-supplementary.pdf]

Supplementary Materials

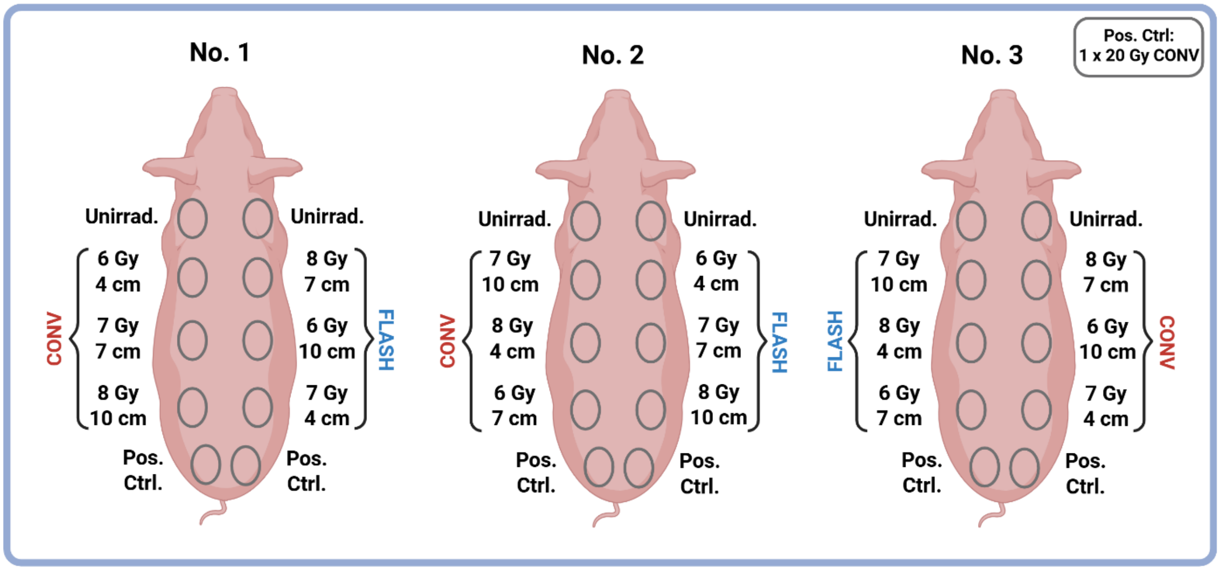

Supplementary Figure S1. Illustration of the field placements on each animal. A total of 18 unique combinations of treatment mode (FLASH or conventional dose rates), field size (4, 7, or 10 cm) and fraction sizes (6, 7, or 8 Gy), were distributed amongst the three animals. In addition, each animal had two paired "positive control" fields (1 × 20 Gy CONV) and two paired unirradiated "negative control" fields. Created in BioRender. Konradsson, E. (2026) https://BioRender.com/ekk51za.

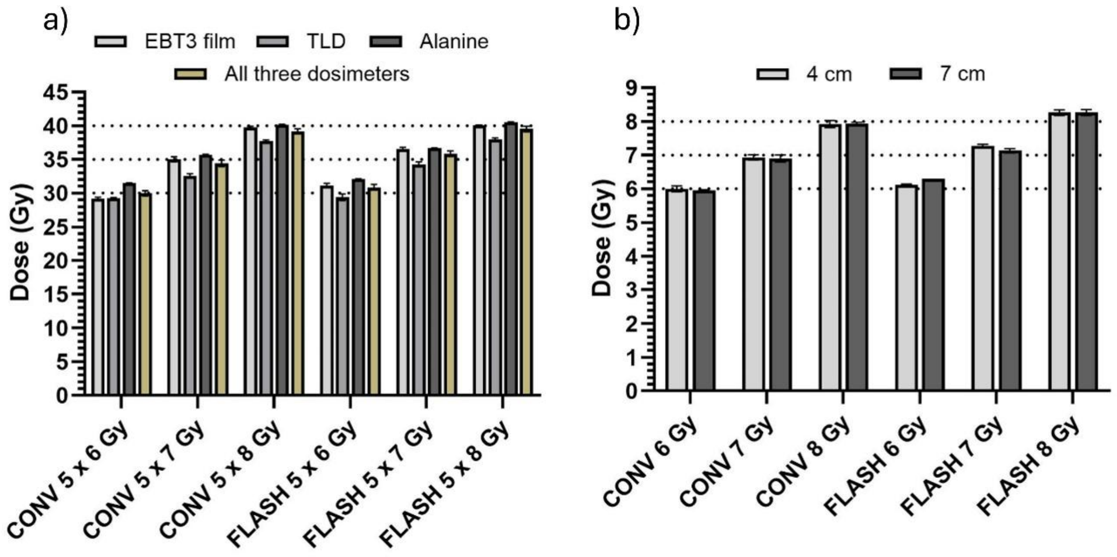

Supplementary Figure S2. Pretreatment dose verification in the prescribed geometry, i.e., at the depth of dose maximum, including skin collimation and 1 cm bolus. (**a**) Doses (given in 5 fractions) to a 10-cm field measured with radiochromic EBT3 film, thermoluminescent dosimeters (TLDs), and alanine. (**b**) Doses (given in 1 fraction) to the 4- and 7-cm fields measured with radiochromic EBT3 film. Results are shown as the mean ± SEM of three repeated measurements. TLD, thermoluminescent dosimeter.

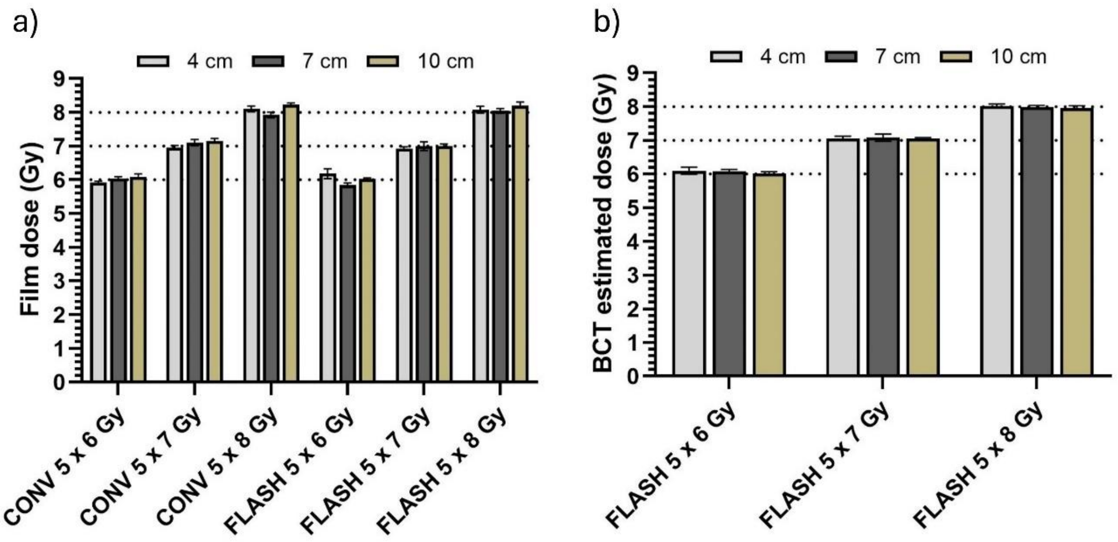

Supplementary Figure S3. In vivo dose validation during animal irradiations. (**a**) Doses measured with EBT3 films placed at the skin surface. Readouts were extrapolated to the prescription depth. (**b**) Doses estimated by the real-time output of the beam current transformer (BCT). Results shows the mean ± SEM from the 5 fractions to each field.

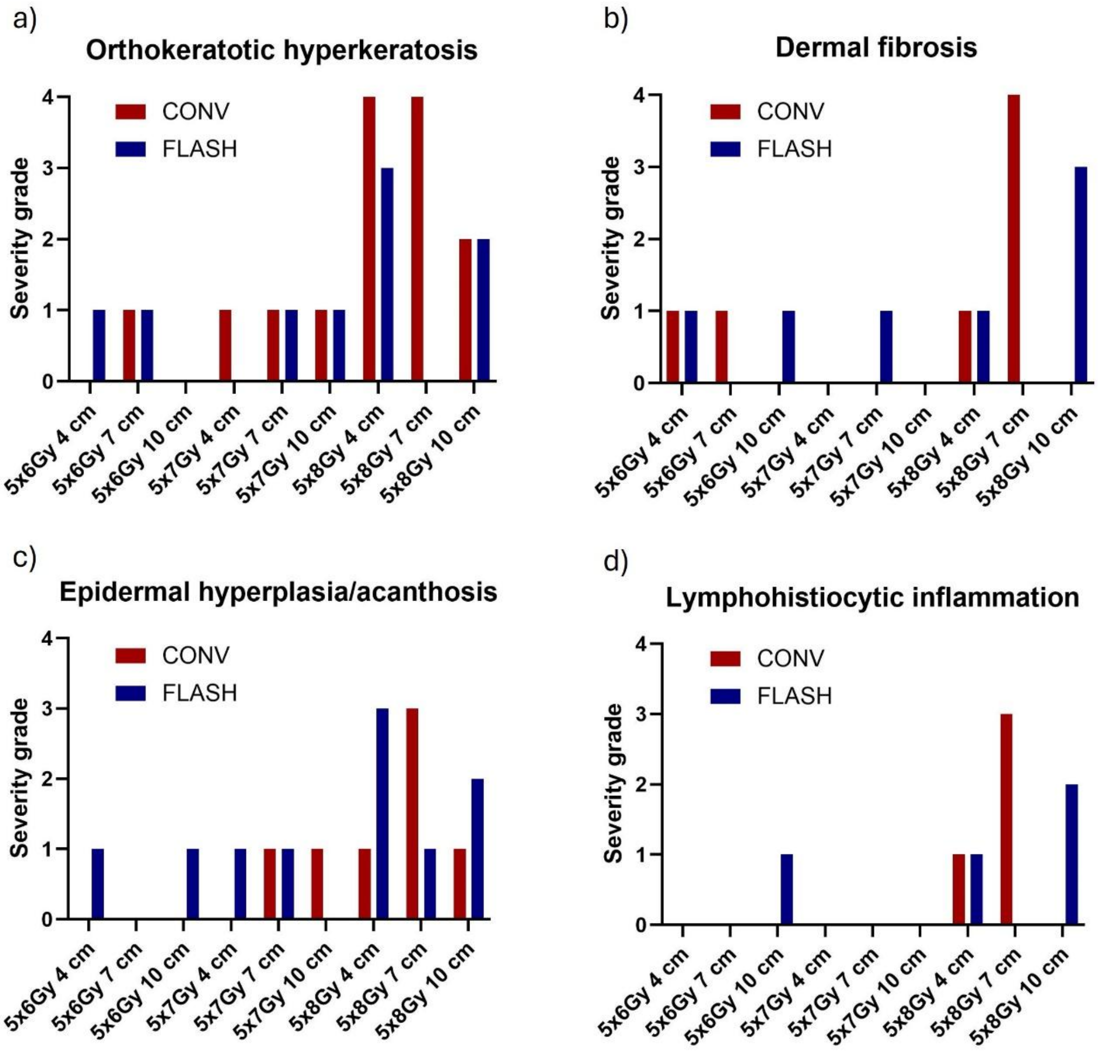

Supplementary Figure S4. Histopathologic evaluation of porcine skin after fractionated conventional (CONV; 8 Gy/min) or FLASH (175-246 Gy/s) irradiation. Tissue samples were collected at the end of follow-up (152–165 days after the final fraction) for histologic analysis. Fields treated with (**a**,**b**) 5 × 8 Gy exhibited up to grade 4 orthokeratotic hyperkeratosis and dermal fibrosis and (**c**,**d**) up to grade 3 epidermal hyperplasia and lymphohistiocytic inflammation. In contrast, all fields receiving 5 × 6 Gy or 5 × 7 Gy showed only grade 0 or 1 histopathologic changes.

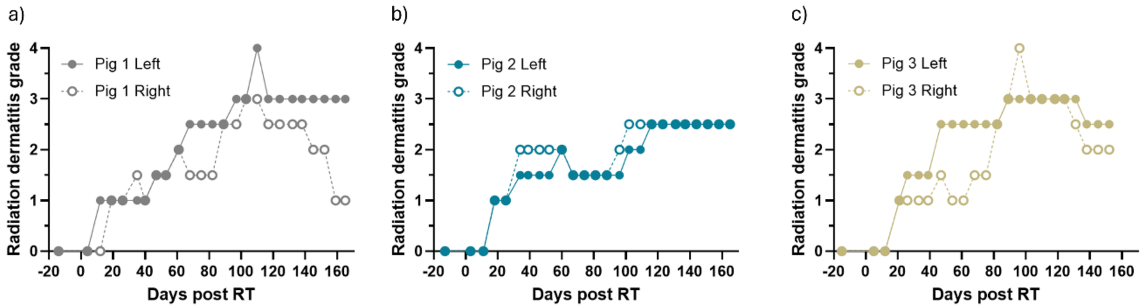

Supplementary Figure S5. (**a**–**c**) Radiation dermatitis graded over time using the modified RTOG radiation dermatitis grading scale (grades 1–4) for each of the six positive control fields (left and right side of each animal) irradiated with 1 × 20 Gy CONV RT (5-cm field size).

Supplementary Table S1. Physical beam parameters used for FLASH and CONV irradiations

Modality	Prescribed total dose, Gy	Prescribed Fx dose, Gy	Field size	PRF, Hz	FWHM, ms, mean ± SD	No. of pulses, mean ± SD	DPP, Gy, mean ± SD	Average dose rate, Gy/s, mean ± SD	Pulse dose rate, MGy/s, mean ± SD
CONV	30	6	4 cm	30	1.2	1241±16	0.005	0.14	0.004
CONV	30	6	7 cm	30	1.2	1360±24	0.004	0.13	0.004
CONV	30	6	10 cm	30	1.2	1275±41	0.005	0.14	0.004
CONV	35	7	4 cm	30	1.2	1686±29	0.004	0.12	0.003
CONV	35	7	7 cm	30	1.2	1599±43	0.004	0.13	0.004
CONV	35	7	10 cm	30	1.2	1729±42	0.004	0.12	0.003
CONV	40	8	4 cm	30	1.2	1698±35	0.005	0.14	0.004
CONV	40	8	7 cm	30	1.2	1784±39	0.004	0.13	0.004
CONV	40	8	10 cm	30	1.2	1991±28	0.004	0.12	0.003
FLASH	30	6	4 cm	90	2.3±0.1	4	1.5±0.07	185±9	0.68±0.02
FLASH	30	6	7 cm	90	2.3±0.1	4	1.5±0.03	175±4	0.63±0.01
FLASH	30	6	10 cm	90	2.6±0.3	4	1.5±0.02	181±2	0.58±0.02
FLASH	35	7	4 cm	90	2.5±0.1	4	1.7±0.03	208±3	0.68±0.02
FLASH	35	7	7 cm	90	2.7±0.1	4	1.8±0.06	210±8	0.64±0.02
FLASH	35	7	10 cm	90	2.9±0.1	4	1.8±0.03	210±4	0.60±0.01
FLASH	40	8	4 cm	90	2.9±0.1	4	2.0±0.05	242±6	0.69±0.01
FLASH	40	8	7 cm	90	3.2±0.1	4	2.0±0.03	241±4	0.63±0.0.2
FLASH	40	8	10 cm	90	3.4±0.1	4	2.0±0.05	246±7	0.60±0.01

Abbreviations: Fx, fractions; PRF, pulse repetition frequency; FWHM, full width half maximum; DPP, dose per pulse; SD, standard deviation.

Supplementary Table S2. Paired comparisons were performed at the animal level using the Wilcoxon signed-rank test (two-sided; Pratt). Effect sizes are reported as Hodges–Lehmann (HL) median within-animal differences (CONV − FLASH) with 95% confidence intervals (CI).

**Dose (Gy)**	**Endpoint**	**HL median difference (CONV − FLASH)**	**95% CI**	**Wilcoxon–Pratt *p***
30 (5×6)	Peak dermatitis (grade)	0.0	−0.5 to 0.5	>0.999
30 (5×6)	Dermatitis at end of study	0.0	0 to 0	N/A
30 (5×6)	Time to progression (weeks)	0.0	−1 to 2	>0.999
30 (5×6)	Time to peak dermatitis (weeks)	−2.0	−5 to 0	0.50
30 (5×6)	Peak erythema index	13.0	−45 to 51	0.75
30 (5×6)	Epidermal thickness (μm)	−1.44	−10.95 to 5.95	0.75
30 (5×6)	Dermal thickness (μm)	−175.1	−390.9 to 195.5	0.75
35 (5×7)	Peak dermatitis (grade)	0.0	−0.5 to 0	>0.999
35 (5×7)	Dermatitis at end of study	0.0	0 to 0	N/A
35 (5×7)	Time to progression (weeks)	0.0	−1 to 0	>0.999
35 (5×7)	Time to peak dermatitis (weeks)	0.0	0 to 1	>0.999
35 (5×7)	Peak erythema index	−12.0	−24 to −4	0.25
35 (5×7)	Epidermal thickness (μm)	−0.51	−7.66 to 0.67	0.75
35 (5×7)	Dermal thickness (μm)	−464.2	−738.8 to 898.6	>0.999
40 (5×8)	Peak dermatitis (grade)	0.0	−1 to 0	>0.999
40 (5×8)	Dermatitis at end of study	0.0	0 to 1.5	>0.999
40 (5×8)	Time to progression (weeks)	0.0	−1 to 0	>0.999
40 (5×8)	Time to peak dermatitis (weeks)	0.0	0 to 1	>0.999
40 (5×8)	Peak erythema index	−50.0	−107 to 42	0.50
40 (5×8)	Epidermal thickness (μm)	7.14	−7.89 to 8.73	0.75
40 (5×8)	Dermal thickness (μm)	4.41	−2533 to 3093	0.75

Dermatitis at end of study was constant (no variability) in some dose groups, so Wilcoxon *p*-values are not applicable (N/A).
